# Effects of 52-Day Oral Exposure to Fluorescent Polystyrene Microplastics on Hormonal Profile, Sperm Parameters, and Fertility in Male Wistar Rats

**DOI:** 10.3390/toxics14040318

**Published:** 2026-04-09

**Authors:** Hristiyana Kanzova, Madlena Andreeva, Yana Goranova, Rosen Ivanov, Stefan Manchev, Hristo Gagov, Iliyana Sazdova, Milena Mishonova, Neli Raikova, Lea Koceva, Dilyana Doncheva-Stoimenova, Pavel Rashev, Albena Alexandrova, Elina Tsvetanova

**Affiliations:** 1 Institute of Neurobiology, Bulgarian Academy of Sciences, Acad. Georgi Bonchev Str., Bl. 23, 1113 Sofia, Bulgaria; hristiyanakanzova@gmail.com (H.K.); lealk@uni-sofia.bg (L.K.); a_alexandrova_bas@yahoo.com (A.A.); 2 Research Laboratory of Military Toxicology, Department of Disaster Medicine and Toxicology, Military Medical Academy, 3 Georgi Sofiyski Str., 1606 Sofia, Bulgaria; yana_goranova@abv.bg; 3 Institute of Experimental Morphology, Pathology and Anthropology with Museum, Bulgarian Academy of Sciences, Acad. Georgi Bonchev Str., Bl. 25, 1113 Sofia, Bulgaria; rosen.ivanov@iempam.bas.bg; 4 Institute of Biology and Immunology of Reproduction “Acad. K. Bratanov”, Bulgarian Academy of Sciences, 73, Tzarigradsko Shose Blvd., 1113 Sofia, Bulgaria; s_manchev@yahoo.co.uk (S.M.); pavel_rashev@abv.bg (P.R.); 5 Department of Animal and Human Physiology, Faculty of Biology, Sofia University “St. Kliment Ohridski”, 8 Dragan Tsankov Blvd., 1164 Sofia, Bulgaria; hgagov@uni-sofia.bg (H.G.); i.sazdova@biofac.uni-sofia.bg (I.S.); mmishonova@biofac.uni-sofia.bg (M.M.); neliraikova@biofac.uni-sofia.bg (N.R.); donchevast@biofac.uni-sofia.bg (D.D.-S.)

**Keywords:** polystyrene microplastics, Wistar rat model, male reproductive function, sperm quality, reproductive hormones, size-dependent effects, fertility potential

## Abstract

Increasing environmental contamination with microplastics (MPs) raises significant concerns regarding their potential impact on reproductive health. This study evaluated the effects of prolonged oral exposure to fluorescent polystyrene microplastics (FPS-MPs) of different sizes on the male reproductive system in Wistar rats. Juvenile male rats at 21 days of age were exposed to FPS-MPs of 1 µm and 5 µm for 52 days, covering the period of sexual maturation and a complete spermatogenesis cycle. Body weight, reproductive organ indices, serum levels of testosterone (T), estradiol (E2), follicle-stimulating hormone (FSH), and luteinizing hormone (LH), as well as sperm motility and morphology, were assessed. Reproductive potential and accumulation of microplastics in reproductive tissues were also evaluated. No significant differences were observed in body weight or most reproductive organ indices, except for a slight reduction in the left epididymis index in the group exposed to 5 µm particles. FPS-MPs induced size-dependent changes in the hormonal profile, including decreases in T and E2 and compensatory increases in FSH and LH, as well as impairments in sperm quality, which were more pronounced in rats exposed to smaller particles. In conclusion, fertility potential remained preserved, while FPS-MPs accumulated in the testes and epididymides, demonstrating subclinical, size-dependent effects on the male reproductive system.

## 1. Introduction

Microplastics (MPs), defined as plastic particles smaller than 5 mm, have emerged as widespread environmental pollutants, detected in aquatic, terrestrial, and atmospheric ecosystems [1,2,3,4]. Early studies highlighted the scale of plastic accumulation and its potential impact on ecosystems [5]. More recent research indicates that MPs can enter the food chain and accumulate in biological systems, raising significant concerns about their effects on human and animal health [6,7]. Due to their small size, unique physicochemical properties, and ability to adsorb environmental contaminants, MPs can act as carriers of toxic substances and biological stressors, potentially disrupting physiological processes, including endocrine regulation and reproductive function [8,9].

Among the various types of MPs, polystyrene microplastics (PS-MPs) are widely used in experimental toxicological studies due to their stability, well-characterized particle sizes, and relevance to realistic exposure scenarios [10,11]. Fluorescent polystyrene microplastics (FPS-MPs) are particularly suitable for laboratory investigations, as they allow tracking of particle uptake, distribution, and bioaccumulation in tissues. Previous studies have demonstrated that MPs can cross biological barriers, including the intestinal epithelium blood–testis barrier, and accumulate in organs such as the liver, kidneys, brain, and reproductive tissues [12,13], raising significant concerns regarding their potential impact on male reproductive health.

Recently published human studies have detected MPs in human semen, with exposure being associated with alterations in certain semen parameters, including reduced progressive sperm motility, raising concerns about potential adverse effects on male fertility [14]. MPs have also been found in other human reproductive fluids, including follicular fluid, highlighting the ability of these particles to reach reproductive tissues and emphasizing the need for further in-depth investigation [15].

Experimental studies in animal models have confirmed that MPs can induce endocrine disruptions by altering the reproductive hormones testosterone (T), luteinizing hormone (LH), and follicle-stimulating hormone (FSH) and dysregulating the hypothalamic–pituitary–gonadal axis [16,17]. These hormonal changes are associated with suppressed spermatogenesis, altered sperm morphology, and decreased sperm motility and viability [16,18,19]. In addition, increased oxidative stress, inflammatory responses, and cellular damage in testicular tissue have been observed, representing a well-established link to male reproductive toxicity [20,21].

The biological effects of MPs depend on particle size, concentration, exposure duration, and the developmental stage of the organism [22]. Smaller particles penetrate tissues more efficiently and can elicit stronger biological responses compared to larger ones [13,23]. Adolescence and puberty represent particularly sensitive periods for reproductive system development, with exposure during this window potentially leading to long-term alterations in endocrine regulation and reproductive function in the adult organism [24]. In rodents, this period corresponds to critical testicular maturation and the progression of spermatogenesis, making it a suitable model for toxicological investigations [25,26].

Despite the growing number of studies, significant knowledge gaps remain regarding the effects of prolonged oral exposure to MPs starting at puberty and spanning a full spermatogenesis cycle. A comprehensive understanding of these effects is essential for assessing the potential risks to male reproductive health under chronic MP exposure.

The aim of this study was to evaluate the effects of a 52-day oral exposure to FPS-MPs of 1 µm and 5 µm on male Wistar rats. Body weight, reproductive organ indices, hormonal profile (T, estradiol (E2), FSH, and LH), sperm quality (motility, kinematic parameters, and morphology), and accumulation of MPs in testicular tissue were analyzed. To assess the impact on fertilization potential, males were paired with females treated under the same conditions, and reproductive success was determined by counting the number of fetuses on day 15 of gestation. This approach allows a reliable and standardized assessment of fertilizing capacity before parturition.

## 2. Materials and Methods

### 2.1. Experimental Design

A total of 36 Wistar rats (18 males and 18 females) aged 21 days were used in the experiment. Animals were housed under standard controlled laboratory conditions (temperature 22 ± 1 °C, relative humidity 50 ± 5%, 12 h light/dark cycle) in standard cages with ad libitum access to food and water. Animals were allowed to acclimate to the laboratory conditions for one week, after which they were randomly assigned to experimental groups. All procedures were conducted in accordance with Directive 2010/63/EU on the protection of animals used for scientific purposes. Animals were supplied by EB-BEA (Slivnitsa, Bulgaria), and the study protocol was approved by the Institutional Ethics Committee (Approval No. 425/24 February 2025).

Male animals (*n* = 18) were randomly assigned to three groups of six animals each:

- Control group (Co♂)—received purified drinking water.
- G1♂—exposed to 1 µm red-fluorescent polystyrene microplastics (FPS-MPs; Magsphere Inc., Pasadena, CA, USA; Cat. No. PSFR001UM).
- G5♂—exposed to 5 µm red-fluorescent polystyrene microplastics (FPS-MPs; Magsphere Inc., Pasadena, CA, USA; Cat. No. PSFR005UM).

The exposure to FPS-MPs was performed via voluntary consumption in drinking water at a fixed dose of 0.1 mg/animal/24 h for 52 consecutive days. The 52-day exposure period was chosen to cover approximately one complete spermatogenic cycle in rats, allowing the assessment of potential effects of MPs on all stages of sperm development and maturation. The suspension was sonicated for 30 min prior to administration to ensure uniform particle distribution. The selected dose of 0.1 mg/animal/day was based on previously published studies investigating the sub-chronic effects of PS-MPs in rodents, providing sufficient exposure to elicit measurable biological responses over a complete spermatogenic cycle without inducing systemic toxicity [27].

To assess male fertilization potential, each male rat was paired with a female rat (*n* = 18) treated under the same experimental conditions (Co♀, G1♀, and G5♀) for the same duration, with each pair consisting of 1♂:1♀. Female rats were included solely to evaluate male reproductive outcomes and were not subjected to additional analyses, as the study was specifically designed to focus on the male reproductive factor.

### 2.2. Body Weight Measurement and Calculation of Organ Indices

The body weight of the animals was measured at the beginning of the experimental period (21 days of age) and at the end of the 52-day exposure (73 days of age). After the final measurement, the rats were anesthetized with ketamine/xylazine (Anaket 100 mg/mL, VetViva Richter GmbH, Wels, Austria; Xylazin 2%, Bioveta a.s., Ivanovice na Hané, Czech Republic) and the testes and epididymides were collected. The weights of the left and right testes, as well as the left and right epididymides, were determined using an analytical balance.

For standardized assessment of organ size relative to body weight, the organ index was calculated using the following formula:

(1) $$\mathrm{Organ}\;\mathrm{index}\;\left(\%\right)=\frac{\mathrm{Organ}\;\mathrm{weight}\;\left(g\right)}{\;\mathrm{Final}\;\mathrm{body}\;\mathrm{weight}\;\left(g\right)\;\;}\times 100$$

where Organ weight is the weight of the specific organ (testis or epididymis), and Final body weight is the animal’s body weight at the end of the exposure period.

The testis and epididymis indices allow for comparison of relative organ mass between experimental groups while accounting for individual differences in body weight.

### 2.3. Hormonal Status

At the end of the exposure period, blood was collected via cardiac puncture and centrifuged at 3500 rpm for 10 min to separate the serum. Hormone levels were determined using the ELISA method with the following kits:

- Rat LH (Luteinizing Hormone) ELISA Kit, 96T (Cat. No. E-EL-R0026), Elabscience, Houston, TX, USA.
- Rat FSH (Follicle Stimulating Hormone) ELISA Kit, 96T (Cat. No. E-EL-R0391), Elabscience, Houston, TX, USA.
- QuicKey Pro Rat Testosterone (T) ELISA Kit, 96T (Cat. No. E-OSEL-R0003), Elabscience, Houston, TX, USA.
- QuicKey Pro Rat Estradiol (E2) ELISA Kit, 96T (Cat. No. E-OSEL-R0001), Elabscience, Houston, TX, USA.

### 2.4. Sperm Assessment

#### 2.4.1. Sperm Motility

Sperm motility and kinematics were assessed using a computerized SCA system (Sperm Class Analyzer 5.0, Microptic, Barcelona, Spain). Samples were collected on day 52 and diluted 1:10 to an appropriate concentration and loaded into a Leja 20 chamber (Leja Products B.V., Nieuw-Vennep, Netherlands). The analysis was performed under a microscope with a heated stage at 37 °C (Nikon, Tokyo, Japan), observing 10 μL of diluted semen across five different fields of view.

The following parameters were recorded using the SCA software:

- Total motility (TM, %)—the percentage of progressive and non-progressive sperm.
- Progressive motility (P, %)—the percentage of sperm exhibiting forward progression.
- Non-progressive motility (NP, %)—the percentage of motile sperm without forward progression.
- Immotile sperm (Static, %)—the percentage of non-motile sperm.

Kinematic parameters:

- VCL (curvilinear velocity, μm/s)—average velocity along the actual trajectory of the sperm head.
- VSL (straight-line velocity, μm/s)—average velocity along a straight line from the starting to the ending position.
- VAP (average path velocity, μm/s)—average velocity along the smoothed path of the sperm head.
- LIN (%)—linearity, ratio of VSL to VCL.
- STR (%)—straightness, ratio of VSL to VAP.
- WOB (%)—wobble, oscillation of the trajectory relative to the average path.

#### 2.4.2. Sperm Morphological Assessment

The morphological assessment of sperm was performed using the SpermBlue Kit (Microptic, Barcelona, Spain). Smears of sperm suspensions were prepared and examined under a light microscope (Olympus BX51, Olympus Corporation, Tokyo, Japan) equipped with an Olympus XC50 camera (Olympus Corporation, Tokyo, Japan). On each smear, 100 spermatozoa were counted, evaluating:

- Morphologically normal and abnormal sperm;
- Specific types of abnormalities in the head, midpiece, and tail.

### 2.5. Reproductive Parameters

To assess the effect of FPS-MPs on male fertilization potential, treated males were paired with females exposed under the same conditions, with each pair consisting of 1♂:1♀. Mating was allowed for 5 consecutive days, after which males were allocated. Pregnancy outcomes were examined by the presence and size of the litter, allowing a reliable and standardized assessment of male fertility prior to the onset of potential prenatal loss or parturition.

The following parameters were recorded:

- Fertility index (%), calculated using the formula:

(2) $$\mathrm{Fertility}\;\mathrm{index}\;\left(\%\right)=\frac{\;\mathrm{number}\;\mathrm{of}\;\mathrm{pregnant}\;\mathrm{females}\;}{\mathrm{number}\;\mathrm{of}\;\mathrm{mated}\;\mathrm{females}}\times \;100$$

- Mean litter size per pregnant female and total number of embryos—to evaluate offspring quantity and quality.

### 2.6. Accumulation of Microplastics

The accumulation of FPS-MPs in the testes and epididymides was assessed using the Pearl Trilogy Small Animal Imaging System (LI-COR Biotechnology GmbH, Bad Homburg, Germany). Particle fluorescence was assessed by exciting with a 680 nm laser and detected at 700 nm. The penetration and distribution of FPS-MPs in the reproductive tissues were evaluated based on fluorescence intensity relative to background. Fluorescence was quantified using region-of-interest (ROI) analysis and expressed as arbitrary fluorescence units (AFU) normalized to background signal. The relative fluorescence signal was standardized by using equal surface of selected area for each image. The images were analysed by Image Studio Software v6.0, Pearl Trilogy system.

### 2.7. Statistical Analysis

Data were analyzed using one-way ANOVA followed by Tukey’s post hoc test. Statistical analyses were performed with IBM SPSS Statistics, version 26 (SPSS Inc., Chicago, IL, USA). Results are presented as mean ± SD. Statistical significance was considered at * *p* ≤ 0.05, ** *p* ≤ 0.01, and *** *p* ≤ 0.001 versus the control group (Co), and † *p* < 0.05, †† *p* < 0.01, and ††† *p* < 0.001 for comparisons between G1 and G5 groups.

### 2.8. Data Availability and Use of AI

All data generated or analyzed during this study are available from the corresponding author upon reasonable request.

Generative artificial intelligence (GenAI) was not used for study design, data collection, analysis, or interpretation. It was only used for minor text editing (grammar, spelling, and formatting).

## 3. Results

The prolonged 52-day exposure to FPS-MPs resulted in significant alterations in multiple parameters related to male reproductive function. Animal body weight, serum hormone levels, sperm motility parameters, morphology and viability, as well as reproductive outcomes following mating, were analyzed. The results are presented in the tables and figures below.

### 3.1. Body Weight and Reproductive Organ Indices

The initial body weight of the animals did not differ significantly between groups (Co: 47.33 ± 2.52 g; G1: 46.67 ± 2.08 g; G5: 48.33 ± 1.53 g). Final body weight after the 52-day exposure also showed no statistically significant differences between the control and experimental groups (Co: 385.33 ± 21.94 g; G1: 377.33 ± 35.92 g; G5: 374.67 ± 32.08 g). The testis index (left and right) was not significantly affected by FPS-MP exposure. The left epididymis index was slightly reduced in G5 compared to the control group (*p* ≤ 0.05), while all other parameters showed no statistically significant differences (Table 1).

### 3.2. Hormonal Profile After FPS-MP Exposure

Serum levels of key reproductive hormones following FPS-MP exposure are presented in Table 2. Analysis of serum hormone levels revealed statistically significant differences between experimental groups for all measured parameters (T, FSH, and LH (*p* ≤ 0.05)), except for E2. E2 levels were reduced in the G1 group (3.98 ± 0.08) compared to the Co group (4.22 ± 0.08; *p* ≤ 0.001), whereas the G5 group (4.10 ± 0.09) did not show a significant difference relative to the Co. T concentrations were significantly decreased in G1 (2.73 ± 0.16; *p* ≤ 0.001) and moderately reduced in G5 (3.13 ± 0.12; *p* ≤ 0.05) compared to the Co group (3.40 ± 0.23). FSH levels were elevated in the exposed groups, with the highest values observed in G1 (3.20 ± 0.09; *p* ≤ 0.001), followed by G5 (3.07 ± 0.08; *p* ≤ 0.001), relative to the Co group (2.58 ± 0.08). A similar trend was observed for LH, where concentrations in G1 (5.18 ± 0.12; *p* ≤ 0.001) and G5 (4.85 ± 0.10; *p* ≤ 0.001) were higher than those in the Co group (4.25 ± 0.10).

### 3.3. Sperm Motility

Sperm motility parameters, assessed via SCA analysis, are presented in Table 3. Significant alterations were observed in the exposed groups. Progressive motility (P, %) was markedly reduced in G1 (14.79 ± 2.04; *p* ≤ 0.001) and G5 (22.80 ± 3.69; *p* ≤ 0.001) compared to the control group (54.23 ± 6.93). Total motility (TM, %) was also decreased in G1 (56.48 ± 7.32; *p* ≤ 0.001) and G5 (64.74 ± 9.58; *p* ≤ 0.001) relative to Co (83.51 ± 5.73). The percentage of immotile sperm (Static, %) was elevated in the exposed groups, reaching 43.52 ± 7.32 in G1 and 35.26 ± 9.58 in G5, compared to 16.49 ± 5.73 in the control group. Significant differences between groups were also observed for the kinematic parameters VSL, VAP, and LIN.

### 3.4. Sperm Morphology

**Figure 1:**
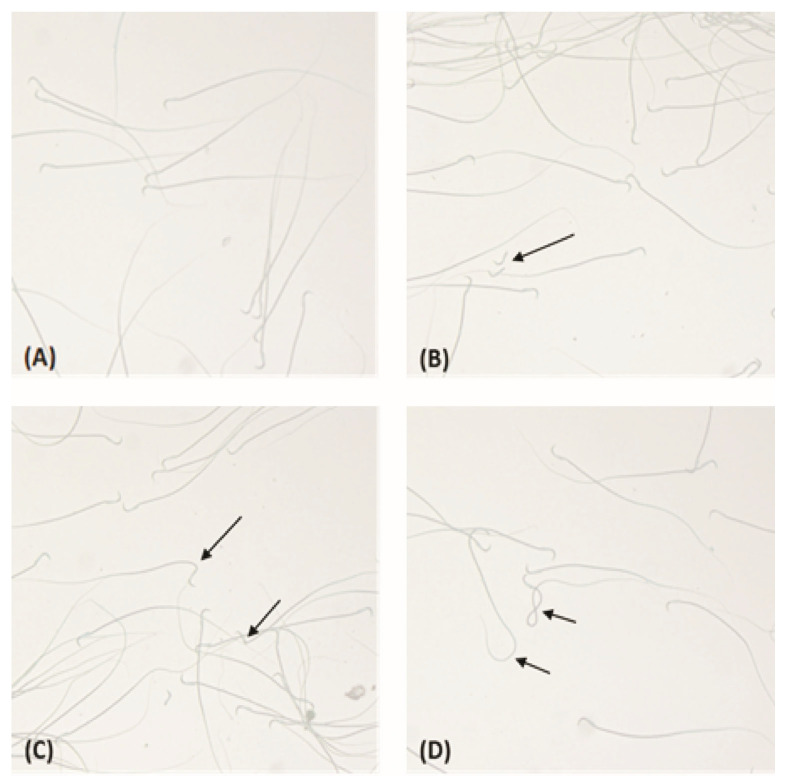
Representative micrographs of rat sperm morphology after exposure to FPS-MPs. Sperm samples were obtained from experimental groups of rats exposed to FPS-MPs with particle sizes of 1 μm and 5 μm and examined under 40× magnification. (**A**) Normal spermatozoa with typical head and intact tail morphology. (**B**) Head abnormalities, including sperm heads without tails. (**C**) Midpiece abnormalities characterized by bending in the midpiece region. (**D**) Tail abnormalities showing coiled or folded tails. Arrows indicate described morphological abnormalities of spermatozoa.

Analysis of sperm morphology revealed statistically significant differences between the control and exposed groups (Table 4), while representative micrographs illustrating the different types of sperm morphological abnormalities are presented in Figure 1. The percentage of morphologically normal sperm was significantly lower in animals exposed to FPS-MPs, with the lowest values observed in the G1 group (81.83 ± 1.60; *p* ≤ 0.001), followed by the G5 group (85.50 ± 0.55; *p* ≤ 0.001), compared to the Co group (92.50 ± 1.64). Accordingly, the overall percentage of abnormal sperm was increased in the exposed groups. Regarding the specific types of morphological abnormalities, a statistically significant increase was observed in head defects in the G1 group (3.83 ± 0.41; *p* ≤ 0.01) and in the G5 group (3.50 ± 0.84; *p* ≤ 0.01) compared to the Co group (1.83 ± 1.17). Tail abnormalities showed the most pronounced changes following microplastic exposure, reaching 11.83 ± 1.47 in the G1 group and 8.33 ± 0.82 in the G5 group (*p* ≤ 0.001 vs. control), with a statistically significant difference also detected between the two exposed groups (*p* ≤ 0.001). For midpiece abnormalities, there was a tendency toward an increase in the exposed groups (G1: 2.50 ± 0.55; G5: 2.67 ± 1.21) compared to the Co group (1.67 ± 0.82), although these differences did not reach statistical significance.

These results indicate that FPS-MP exposure is associated with an increased frequency of sperm morphological abnormalities, with the most pronounced changes observed in the tail structure.

### 3.5. Reproductive Performance

Differences in reproductive outcomes were observed among the experimental groups (Table 5). In the control group and G5 group, all females became pregnant (6/6; fertility index 100%), whereas in G1, pregnancy was confirmed in 5 out of 6 females (83.3%). The mean number of embryos per pregnant female showed a slight tendency to decrease in the exposed groups compared to the control (Co: 11.40 ± 1.52; total embryos: 57; G1: 10.50 ± 2.08; total embryos: 42; G5: 9.60 ± 1.52; total embryos: 48). These data suggest a potential effect of FPS-MPs on reproductive outcomes, although no statistically significant differences were observed between the groups.

### 3.6. Detection of Microplastics in Testicular Tissue

**Figure 2:**
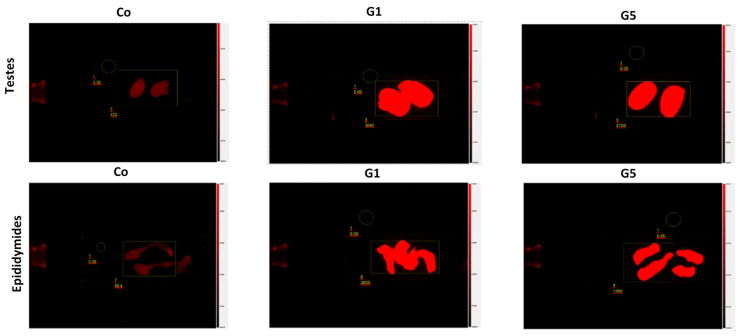
Representative fluorescence images of FPS-MP accumulation in testicular and epididymal tissues of rats after 52-day exposure, presented as fluorescence intensity relative to background.

Analysis of FPS-MP accumulation revealed statistically significant differences between the control and exposed groups (Table 6), while representative fluorescence images illustrating accumulation in testicular and epididymal tissues are presented in Figure 2. No specific fluorescent signal was observed in the control group, with measured intensity remaining low and close to background levels (testicular tissue: 122.00 ± 16.86 AFU; epididymis: 82.07 ± 19.79 AFU). In the exposed groups, fluorescence intensity was significantly higher, with the highest values observed in G1 (testicular tissue: 3597.00 ± 154.89 AFU; epididymis: 2590.50 ± 121.63 AFU; *** *p* ≤ 0.001 vs. Co) and lower values in G5 (testicular tissue: 2600.00 ± 187.08 AFU; epididymis: 1393.33 ± 107.83 AFU; *** *p* ≤ 0.001 vs. Co; ††† *p* ≤ 0.001 vs. G1).

These results indicate that FPS-MP accumulation in male reproductive tissues is size-dependent, with smaller particles (G1) showing higher levels of tissue accumulation than larger particles (G5), while the control group shows minimal background fluorescence.

## 4. Discussion

The increasing presence of MPs in the environment raises significant concerns regarding their potential impact on reproductive health in both animals and humans. The present study provides an integrated analysis of the effects of prolonged 52-day oral exposure to FPS-MPs of different sizes on the male reproductive system of Wistar rats, allowing for the assessment of hormonal profiles, sperm parameters, morphological alterations, fertilization potential and accumulation of MPs in reproductive tissues. Regarding body weight and reproductive organ indices, no statistically significant differences were observed between the Co and experimental groups, except for a slightly reduced left epididymis index in the 5 µm experimental group. In contrast, some experimental studies in rats exposed to PS-MPs have reported significant alterations in body weight and reproductive organ indices, including increased body weight gain and reduced relative weights of the testes and epididymis, accompanied by an increased prostate index. These differences may reflect variations in exposure conditions such as particle size, dose, and duration of treatment [28]. While other experimental studies in mice have reported significant reductions in body weight following exposure to PS-MPs [29], such effects were not observed in the present study. The reduction in body weight reported in mouse studies has been linked to the organism’s difficulty in eliminating larger MPs, which increases gastrointestinal burden and may reduce nutrient absorption [16]. In the rats of the present study, the lack of significant changes is likely due to their higher resilience to subchronic doses or to differences in particle size and concentration, which limit accumulation and gastrointestinal stress, thereby preserving nutrient absorption.

Exposure induced size-dependent changes in serum levels of T, E2, FSH, and LH, with smaller particles (1 µm, G1) causing more pronounced alterations, while larger particles (5 µm, G5) led to moderate changes. Smaller FPS-MPs exhibit higher bioavailability and can cross the blood–testis barrier, accumulating in the testes and potentially compromising Leydig cell function [12,13,30,31]. Recent studies also demonstrated that orally administered MPs of various sizes can directly enter Leydig, Sertoli, and germ cells, bioaccumulating in testicular tissue and altering spermatogenesis, sperm morphology, and testosterone biosynthesis [16,32]. The reduced T levels, accompanied by elevated gonadotropins, indicate a compensatory activation of the hypothalamic–pituitary–gonadal axis in response to partially impaired testicular function, likely driven by oxidative stress, mitochondrial dysfunction, and disrupted enzymatic activity in the testes [33,34,35]. Similar hormonal patterns have been reported in experimental studies with PS-MPs in rats, where exposure resulted in decreased testosterone levels accompanied by increased LH and FSH, suggesting disruption of the hypothalamic–pituitary–gonadal axis and compensatory endocrine responses to testicular dysfunction [28].

Sperm motility, a key indicator of fertilization potential, was significantly reduced following FPS-MP exposure, with the effect being more pronounced for smaller particles (1 µm), indicating that even subchronic doses can impair sperm locomotor activity. The reduction in motility corresponds with effects observed in other laboratory animal models, typically associated with higher doses, longer exposure periods, or concomitant oxidative stress and testicular damage [27,29,36,37]. In human studies, the impact on total motility is generally less pronounced, although specific parameters, such as progressive motility, can be affected depending on the polymer type [14]. It is important to note that in the present study, spermatozoa were isolated directly from the *ductus deferens* rather than from ejaculate, which may partly explain the lower motility values and reflect stages of functional maturation.

Morphological analysis revealed structural alterations, including a reduction in the proportion of morphologically normal sperm and an increase in abnormal forms, suggesting that FPS-MPs influence spermatogenesis and cellular differentiation processes. The observed anomalies affected the head, midpiece, and tail, supporting the potential impact of MPs on sperm formation and structural organization. Morphological defects are considered a sensitive indicator of testicular function, as MPs can induce inflammation, cellular stress, and structural changes within the seminiferous tubules [16,27,38]. Moreover, MPs can act as carriers for environmental contaminants such as phthalate esters, and co-exposure increases oxidative stress and transcriptome alterations, further impairing spermatogenesis and sperm quality [39]. Similar findings have been reported in other PS-MP exposures, associated with increased oxidative stress and impaired sperm quality [40,41,42].

Despite the observed hormonal and morphological changes, the fertilization potential remained preserved, as the fertility index and the number of embryos did not differ significantly between the control and experimental groups. This contrasts with data from mice, where MP exposure has been associated with reduced implantation numbers, increased embryonic resorption, and fetal abnormalities [43,44,45], likely reflecting species-specific resilience in rats. The maintained fertility under the present conditions suggests that the observed hormonal and morphological alterations may represent early subclinical effects, highlighting the need for long-term and multigenerational studies.

Fluorescence analysis confirmed the presence of FPS-MPs in testicular and epididymal tissues, demonstrating systemic distribution and tissue retention of the particles following oral exposure. This indicates that MPs can cross the intestinal barrier and accumulate in peripheral organs, including reproductive tissues [30,46,47]. Smaller particles exhibit higher bioavailability and greater potential for interaction with cellular membranes, which corresponds to the more intense fluorescent signal observed with 1 µm FPS-MPs [48]. The accumulation of particles is biologically relevant, as it demonstrates the capacity of FPS-MPs to reach and persist in reproductive tissues, which may be associated with the observed alterations in spermatogenesis, sperm morphology, and hormonal profiles. In addition, the significantly increased accumulation observed in reproductive tissues after sexual maturation likely indicates not only a size- or dose-dependent distribution of FSP-MPs across organs but also a metabolic association. This relationship is indirect due to increased blood flow and lipid content.

## 5. Conclusions

Our 52-day oral exposure to FPS-MPs in Wistar rats suggests size-dependent alterations in the male reproductive system. Subchronic exposure was associated with decreased serum levels of T and E2, accompanied by increased FSH and LH. Sperm motility and the proportion of morphologically normal cells were reduced, while the frequency of head, midpiece, and tail abnormalities increased, with more pronounced effects observed for the smaller particles (1 µm). FPS-MPs were detected in testicular and epididymal tissues, yet fertilization potential remained preserved, indicating that these exposures induce subclinical, size-dependent effects on hormonal balance and sperm function without compromising overall fertility under the conditions of the present study. These findings highlight that, although measurable effects occur at the tissue and cellular levels, overall reproductive capacity remains intact in this experimental model, while future studies will explore longer-term, systemic, and mechanistic effects of microplastic exposure.

## Figures and Tables

**Table 1:** Body weight and reproductive organ indices in control and FPS-MPs-treated rats after 52 days of exposure. Values are presented as mean ± SD (*n* = 6 animals per group).

Parameter	Co	G1	G5
Initial body weight (g)	47.33 ± 2.52	46.67 ± 2.08	48.33 ± 1.53
Final body weight (g)	385.33 ± 21.94	377.33 ± 35.92	374.67 ± 32.08
Testis index L (%)	0.42 ± 0.03	0.43 ± 0.03	0.40 ± 0.01
Testis index R (%)	0.41 ± 0.02	0.41 ± 0.04	0.40 ± 0.01
Epididymis index L (%)	0.16 ± 0.01	0.15 ± 0.01	0.14 ± 0.01 *
Epididymis index R (%)	0.14 ± 0.03	0.16 ± 0.02	0.15 ± 0.01

* *p* ≤ 0.05 vs. Co group.

**Table 2:** Serum hormone levels in rats after exposure to fluorescent polystyrene microplastics (FPS-MPs). Data are presented as mean ± SD (*n* = 6 animals per group).

Parameters	Co	G1	G5
E2	4.22 ± 0.08	3.98 ± 0.08 ***	4.10 ± 0.09
T	3.40 ± 0.23	2.73 ± 0.16 ***	3.13 ± 0.12 * †
FSH	2.58 ± 0.08	3.20 ± 0.09 ***	3.07 ± 0.08 *** †
LH	4.25 ± 0.10	5.18 ± 0.12 ***	4.85 ± 0.10 *** †

* *p* ≤ 0.05, *** *p* ≤ 0.001 versus Co group. † *p* ≤ 0.05 between G1 and G5 groups.

**Table 3:** Sperm motility parameters assessed by SCA after 52-day exposure to FPS-MPs. Data are presented as mean ± SD (*n* = 6 animals per group).

Parameters	Co	G1	G5
Progressive motility (P, %)	54.23 ± 6.93	14.79 ± 2.04 ***	22.80 ± 3.69 *** ††
Non-progressive motility (NP, %)	29.28 ± 7.95	41.69 ± 7.96 **	41.94 ± 6.41 **
Total motility (TM, %)	83.51 ± 5.73	56.48 ± 7.32 ***	64.74 ± 9.58 *** †
Immotile sperm (Static, %)	16.49 ± 5.73	43.52 ± 7.32 ***	35.26 ± 9.58 *** †
VCL (µm/s)	105.60 ± 25.43	92.58 ± 4.28	95.29 ± 14.59
VSL (µm/s)	21.32 ± 5.05	17.80 ± 4.95	25.01 ± 2.29 ††
VAP (µm/s)	43.47 ± 2.38	39.15 ± 4.31	45.08 ± 8.22 †
LIN (%)	27.16 ± 5.12	22.89 ± 3.88	28.97 ± 5.57 †
STR (%)	47.20 ± 6.56	45.71 ± 9.36	52.05 ± 7.55
WOB (%)	42.60 ± 7.25	43.22 ± 4.85	47.20 ± 4.65

** *p* ≤ 0.01, *** *p* ≤ 0.001 vs. Co group; † *p* ≤ 0.05, †† *p* ≤ 0.01 between G1 and G5.

**Table 4:** Sperm morphology after 52-day exposure to FPS-MPs. Data are presented as mean ± SD (*n* = 6 animals per group).

Parameters	Co	G1	G5
Normal (%)	92.50 ± 1.64	81.83 ± 1.60 ***	85.50 ± 0.55 *** †††
Abnormal (%)	7.50 ± 1.64	18.17 ± 1.60 ***	14.50 ± 0.55 *** †††
Head (%)	1.83 ± 1.17	3.83 ± 0.41 **	3.50 ± 0.84 **
Midpiece (%)	1.67 ± 0.82	2.50 ± 0.55	2.67 ± 1.21
Tail (%)	4.00 ± 1.10	11.83 ± 1.47 ***	8.33 ± 0.82 *** †††

** *p* ≤ 0.01, *** *p* ≤ 0.001 vs. Co group. ††† *p* ≤ 0.001 between G1 and G5 groups.

**Table 5:** Pregnancy and litter outcomes of females after mating with FPS-MP-exposed males. Data are presented as mean ± SD (*n* = 6 females per group).

Group	Fertility Index (%)	Mean Litter Size ± SD	Total Embryos
Co	100	11.40 ± 1.52	57
G1	83.3	10.50 ± 2.08	42
G5	100	9.60 ± 1.52	48

Fertility index (%) was calculated as described in Section 2.

**Table 6:** Accumulation of FPS-MPs in testicular and epididymal tissues. Data are presented as mean ± SD (*n* = 6 animals per group).

Group	Co	G1	G5
Testicular tissue	122.00 ± 16.86	3597.00 ± 154.89 ***	2600.00 ± 187.08 *** †††
Epididymis	82.07 ± 19.79	2590.50 ± 121.63 ***	1393.33 ± 107.83 *** †††

*** *p* ≤ 0.001 vs. Co group. ††† *p* ≤ 0.001 between G1 and G5 groups.

## Data Availability

The datasets analyzed and generated during the current study are available from the corresponding author on reasonable request.

## Abbreviations

The following abbreviations are used in this manuscript:

AFU	Arbitrary Fluorescence Units
Co	Control group
E2	Estradiol
EB-BEA	Experimental Breeding Base for Laboratory Animals, Bulgarian Academy of Sciences
FPS-MPs	Fluorescent Polystyrene Microplastics
FSH	Follicle-Stimulating Hormone
G1	Group exposed to 1 µm FPS-MPs
G5	Group exposed to 5 µm FPS-MPs
LH	Luteinizing Hormone
LIN	Linearity, ratio of VSL to VCL
MPs	Microplastics
NP	Non-Progressive Motility
P	Progressive Motility
PS-MPs	Polystyrene Microplastics
ROI	Region of Interest
SCA	Sperm Class Analyzer
STR	Straightness, ratio of VSL to VAP
T	Testosterone
TM	Total Motility
VAP	Average path velocity
VCL	Curvilinear Velocity
VSL	Straight-line velocity
WOB	Wobble, oscillation of the trajectory relative to the average path
